# Zika Virus Pathogenesis: From Early Case Reports to Epidemics

**DOI:** 10.3390/v11100886

**Published:** 2019-09-21

**Authors:** Ryan D. Pardy, Martin J. Richer

**Affiliations:** 1 Department of Microbiology and Immunology, McGill University, Montreal, QC H3A 2B4, Canada; ryan.pardy@mail.mcgill.ca; 2 Rosalind & Morris Goodman Cancer Research Centre, McGill University, Montreal, QC H3G 1Y6, Canada

**Keywords:** Zika virus, pathogenesis, Guillain-Barré syndrome, fetal microcephaly, congenital Zika syndrome

## Abstract

For the first 60 years following its isolation, Zika virus (ZIKV) remained a relatively poorly described member of the *Flaviviridae* family. However, since 2007, it has caused a series of increasingly severe outbreaks and is now associated with neurological symptoms such as Guillain-Barré syndrome and congenital Zika syndrome (CZS). A number of reports have improved our understanding of rare complications that may be associated with ZIKV infection in adults, the areas of the body to which it spreads, and viral persistence in various tissues. Likewise, studies on the effect of ZIKV infection during pregnancy have identified risk factors for CZS and the impact this syndrome has on early childhood. Understanding these outcomes and the factors that drive ZIKV pathogenesis are key to developing vaccination and therapeutic approaches to avoid these severe and potentially debilitating symptoms.

## 1. Introduction

Zika virus (ZIKV) was first isolated from a sentinel rhesus macaque in 1947 in the Ziika forest (more commonly known as the Zika forest) region of Uganda [1]. Although it was presumed to be endemic to the region based on the presence of anti-ZIKV antibodies identified during surveys of human sera [2], the first undisputed account of human infection was not published until 1964 [3]. This and a handful of isolated case reports represented the breadth of the ZIKV literature for the remainder of the 20th century [4,5]. However, ZIKV has since emerged as the cause of a series of increasingly severe outbreaks. Over the course of four months in 2007, 73% of the population of Yap Island, Federated States of Micronesia, was estimated to have been infected with ZIKV [6]. This was followed in 2013 by approximately 32,000 suspected cases of ZIKV infection between October 2013 and April 2014 in French Polynesia [7]. More recently, the 2015–2016 ZIKV epidemic began in Brazil before spreading rapidly throughout South and Central America, with evidence of ZIKV transmission in 84 countries worldwide [8]. In the Americas alone, over 800,000 suspected and confirmed cases were reported over the course of the epidemic [8]. Together, these contemporary outbreaks represent a dramatic change in phenotype for ZIKV, from an innocuous virus that caused isolated infections to one with the potential to cause widespread disease.

In addition to its novel epidemic capacity, these outbreaks have also highlighted the association of neurological symptoms with ZIKV infection. In adults, the primary neurological condition associated with ZIKV infection is Guillain-Barré syndrome (GBS), an acute peripheral neuropathy that causes ascending paralysis. The link between ZIKV infection and GBS was first recognized during the French Polynesian outbreak and was documented in several countries in the South and Central American outbreak that followed [9,10,11,12,13,14]. There are at least three subtypes of GBS: acute inflammatory demyelinating polyradiculoneuropathy (AIDP), acute motor axonal neuropathy (AMAN), and acute motor and sensory axonal neuropathy (AMSAN). AIDP features mononuclear cell (macrophage or T cell) infiltration into the peripheral nervous system (PNS) and destruction of myelin sheaths. For AMAN and AMSAN, pathology is limited to motor neurons or motor and sensory neurons, respectively. Pathology in these subtypes occurs when antibodies against gangliosides on the axon’s plasma membrane cause macrophages to insert between the axon and Schwann cell, leaving the myelin sheath intact but blocking conduction [15]. Although plasma therapy and intravenous immunoglobulin (Ig) treatment improve GBS prognosis, approximately 25% of patients require mechanical ventilation, up to 20% retain some disability one year following the disease, and between 4–15% die from GBS [15]. Thus, the identification of ZIKV as a possible trigger of GBS represents a dangerous new symptom for this typically innocuous virus.

The impact of ZIKV infection during pregnancy and on neonates is the most dramatic aspect of contemporary ZIKV outbreaks. Fetal microcephaly, which is typically diagnosed based on a head circumference at birth that is greater than two standard deviations below average for gestational age and sex, was first associated with ZIKV infection during the South and Central American outbreak [4,16]. Following this observation, retrospective analyses of the French Polynesian outbreak provided further evidence for an association between ZIKV infection during pregnancy and fetal microcephaly [17]. Fetal microcephaly represents one of several symptoms that are collectively referred to as congenital Zika syndrome (CZS), which also includes thin cerebral cortices with subcortical calcifications, macular scarring, and marked early hypertonia [18]. CZS can have lifelong implications for both the affected infant as well as its family. Together, GBS and CZS demonstrate how ZIKV has emerged as a global health threat with the potential to cause severe pathology during, or after, infection.

In this review, we will describe typical and rare outcomes of ZIKV infection in adults, including the areas of the body in which ZIKV has been detected, and the duration of virus detection. We will also examine the effect of ZIKV infection during pregnancy, risk factors for the development of CZS, and the impact of maternal ZIKV exposure on early childhood development. Finally, we will discuss pediatric cases of ZIKV infection, and how the course of infection in children compares to adult cases. Improving our understanding of the severe symptoms that can follow ZIKV infection, and the risk factors associated with them is an important step in order to develop approaches to treat or prevent these symptoms.

## 2. Pathogenesis in Adults

Prior to the Yap Island outbreak, there were very few descriptions of ZIKV infection duration and pathogenesis in humans. The first undisputed account of ZIKV infection in humans was described by a researcher working in the Zika forest in 1964 [3]. In this report of his own infection with ZIKV, Simpson described an acute, five-day illness. During the first two days of infection, he developed a frontal headache, muscle pain, fever, and a diffuse maculopapular rash on his face, neck, trunk, and limbs. On the evening of the second day, his fever broke, and the rash faded over the following three days [3]. Similar to this account, a researcher with a laboratory-acquired ZIKV infection described rapid-onset disease with fever, sweating, retro-orbital pain, and joint pain on the first day, although he reported no rash throughout his illness [19]. Symptoms were maintained for two days and improved until resolution seven days post-onset of symptoms (POS) [19]. Even 36 years later, after ZIKV caused its first major outbreak on Yap Island, the symptoms described were relatively similar: acute rash, fever, arthritis, arthralgia and conjunctivitis, with possible headache, retro-orbital pain, and vomiting [6]. These reports suggest that up until the French Polynesian outbreak, ZIKV caused only a mild, febrile illness with no neurological involvement. However, given the small sample size and lack of control patients, it cannot be definitively stated that neurological symptoms arose only after the Yap Island outbreak.

**Figure 1:**
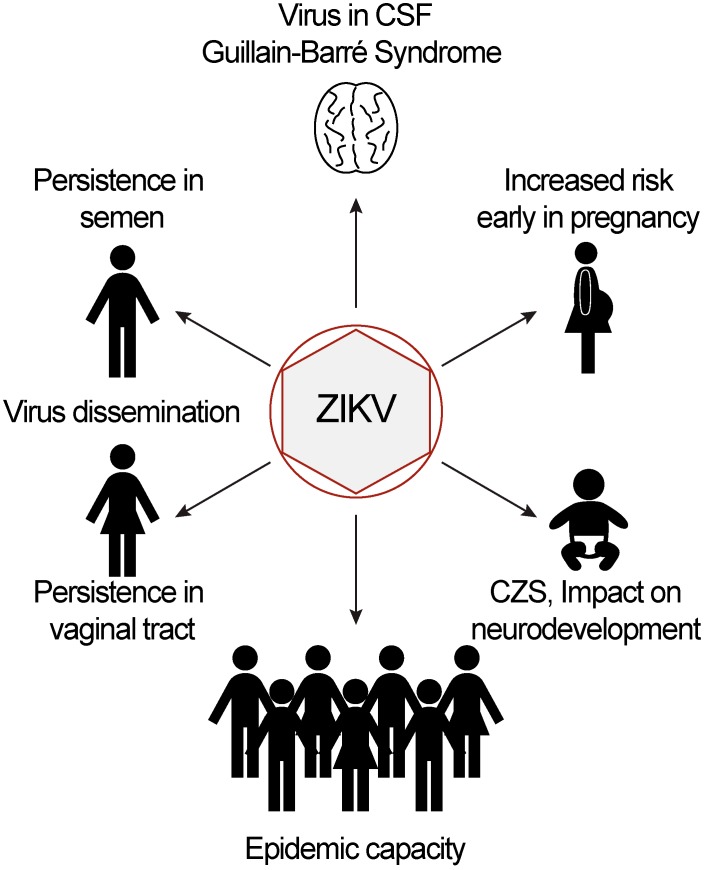
Novel aspects of contemporary Zika virus (ZIKV) outbreaks. Recent ZIKV outbreaks have highlighted the novel epidemic capacity of the virus. Furthermore, ZIKV has been shown to disseminate to many sites throughout the body and ZIKV RNA has been persistently detected long after infection in semen and in the vaginal tract. Most strikingly, ZIKV has been associated with Guillain-Barré syndrome and congenital Zika syndrome (CZS), with impacts on neurodevelopment occurring even in the absence of symptoms like fetal microcephaly. The risk for CZS is increased when infection occurs early during pregnancy. Abbreviations: CSF, cerebrospinal fluid; CZS, congenital Zika syndrome; ZIKV, Zika virus.

In addition to the classic symptoms of ZIKV infection described above, the most common neurological symptom associated with ZIKV infection in adults is GBS (Figure 1). This link was first described during the French Polynesian outbreak in 2013, in a case-control study comparing patients with GBS (cases), patients with a non-febrile illness at the same hospital (control group 1), and ZIKV patients with no neurological symptoms (control group 2) [10]. All GBS cases exhibited generalized weakness, the majority were unable to walk, and mechanical ventilation was required for 12/42 patients [10]. Significantly more ZIKV patients with GBS had IgM antibodies against ZIKV (93%) than control group 1 patients, while ZIKV was detected in 100% of control group 2 patients, providing evidence for ZIKV as a risk factor for GBS [10]. During the South and Central American outbreak, ZIKV was confirmed as a potential trigger for GBS [20], with reports stemming from many countries, including Brazil, Colombia, Honduras, Nicaragua, and Mexico [9,13,14,21,22,23,24]. Reported symptoms were similar to the French Polynesian study, and almost all patients had evidence of ZIKV infection based on either detection of ZIKV RNA or antibodies against ZIKV, in addition to a recent ZIKV-like illness. Where reported, GBS onset tended to be parainfectious, with no asymptomatic period between ZIKV disease and GBS onset [9,13,14,23,24,25]. Additionally, while 25–33% of patients required admission to the intensive care unit (ICU), only three deaths were reported (due to respiratory failure and sepsis) among all GBS cases [21,22,24]. When follow-up with patients occurred after hospital discharge, it was reported that almost all patients recovered successfully from all motor deficits, although in some cases this took as long as six months to one year, and others retained some level of disability at the end of the study [9,21,22,23,24]. Interestingly, GBS associated with ZIKV infection was not restricted to a single subtype across both outbreaks. During the French Polynesian outbreak, nerve conduction studies suggested GBS was most commonly of the AMAN subtype [10]. The AMAN and AMSAN subtypes were also reported in Brazil, Colombia, and Honduras, however, it was most common for GBS to be of the AIDP subtype, which could suggest a different mechanism of pathogenesis during the South and Central American outbreak [13,14,21,22,24]. Nonetheless, these reports identify GBS as a rare but consistent and dangerous potential outcome of ZIKV infection since the French Polynesian outbreak.

In addition to GBS, there have been a variety of case reports and case series describing other rare outcomes that may be associated with ZIKV infection. In one study from a hospital in Rio de Janeiro, Brazil, these included other neurological symptoms such as encephalitis, transverse myelitis, and chronic inflammatory demyelinating polyneuropathy, which is considered to be a chronic version of GBS [21]. Other case studies have identified: meningoencephalitis and meningitis, with ZIKV RNA in the cerebrospinal fluid (CSF; Figure 1) [23,26]; uveitis, with ZIKV RNA in the aqueous humor [27]; and gastrointestinal symptoms associated with infection [28]. In men, other rare but potentially serious symptoms may include a burning sensation during urination or ejaculation, testicular pain, and hematospermia (blood in the semen) [29,30]. Symptoms such as hematospermia occurred only in a small fraction of men who tested positive for ZIKV RNA in their semen, however in a separate study, it was also observed that the presence of ZIKV RNA correlated with lower semen volume and reduced sperm count and motile sperm count [30,31]. Fatal cases of ZIKV infection are exceedingly rare, with only 84 fatal ZIKV cases reported during the entirety of the South and Central American ZIKV outbreak, out of over 800,000 reported cases [8,32]. Thus, in addition to causing the GBS, ZIKV appears to be capable of infecting and causing pathology in numerous areas of the body (Figure 1).

In recent outbreaks, ZIKV has been detected in a wide variety of bodily fluids, including blood, urine, CSF, saliva, semen, vaginal secretions, cervical mucus, and breast milk (Figure 1) [9,21,29,31,33,34,35]. Sexual transmission has been documented for ZIKV [36,37], however there is currently no evidence to suggest that transmission via blood transfusion, saliva, or breastfeeding may occur. Several studies that have examined ZIKV dissemination also analyzed clearance of the virus from bodily fluids over time. In many cases, ZIKV is rapidly cleared, in line with the acute infection most patients experience. However, prolonged presence of ZIKV RNA has been documented in a number of cases (Figure 1). In one study of ZIKV patients in the United States (96% acquired ZIKV infection in other countries), ZIKV RNA was detected as late as 13 days POS in vaginal secretions, 34 days POS in semen, 36 days POS in urine, and in one case, 176 days POS in whole blood [29]. Further, Paz-Bailey and colleagues found in their prospective cohort study of 252 ZIKV patients that the median time to clearance of viral RNA was 15 days POS in serum, 11 days in urine, and 42 days in semen [38]. However, even 90 days POS, 11% of male patients still had ZIKV RNA in their semen [38]. Given the possibility of sexual transmission, several other studies have understandably focused on ZIKV RNA persistence in the female and male genital tracts. Similar to the American study above, two case reports identified ZIKV RNA in vaginal or cervical mucus swabs until 14 and 11 days POS, respectively [34,39]. In men, ZIKV RNA has been detected in semen over 100 days POS, and in one case was detected as late as 240 days POS [30,31,40,41]. One study detected ZIKV antigens in spermatozoa, which provides a potential mechanism for sexual transmission [41]. Factors that are associated with a longer time to clearance of ZIKV RNA from semen, include increased age, presence of conjunctivitis, and less frequent ejaculation, as men who ejaculated four times per week cleared ZIKV 21 days earlier than those who ejaculated once per week [30]. These results must be interpreted with a degree of caution, as the presence of ZIKV RNA does not guarantee or necessarily indicate the presence of an infectious virus. Indeed, Mead et al. were only able to culture ZIKV from semen samples within the first few weeks POS, and only when greater than 10^7^ copies of ZIKV RNA were detected per milliliter of semen [30]. This is consistent with cases of sexual transmission of ZIKV, all of which occurred within the first 41 days POS, with most occurring within the first 20 days [42,43,44,45,46,47]. Thus, although the detection of ZIKV RNA in bodily fluids may overstate the possibility of transmission, the significance of its persistent detection remains to be fully elucidated.

## 3. Pathogenesis During Pregnancy, Infancy, and Childhood

The shocking link between ZIKV infection and pathogenesis during pregnancy first came to light early during the South and Central American outbreak, when a cluster of 58 cases of fetal microcephaly were reported in a single month in Northeastern Brazil, compared to an average of only nine cases per year over the previous four years [48]. Since then, improving our understanding of ZIKV-induced fetal microcephaly and CZS has been a primary focus of ZIKV research. ZIKV-specific IgM has been detected in the CSF of neonates with fetal microcephaly, which is indicative of central nervous system (CNS) infection, and suggests that infection of the developing fetus may be required for fetal microcephaly to develop, not just infection of the mother [49]. Fetal microcephaly and other negative fetal outcomes are most closely linked with maternal ZIKV infection early in pregnancy (first trimester), with one study finding that the risk of below-average neurodevelopment fell by 46% each trimester (Figure 1) [16,50,51,52,53]. In a case-control study of factors associated with the development of CZS, no link was found with maternal age, previous dengue virus infection, or exposure to alcohol, drugs, or tobacco [50]. However, a separate study observed that women who gave birth to microcephalic infants had significantly higher serum neutralizing activity and antibodies reactive to the ZIKV envelope protein domain III, compared to control women [54]. Other birth defects linked to ZIKV infection include patent foramen ovale (failure of a natural hole between the left and right atria of the heart to close before birth), clubfoot, cryptorchidism (failure of the testes to descend into the scrotum), ophthalmologic problems, hearing abnormalities, and difficulty swallowing [16,50,51,52]. In some cases, fetal death following maternal ZIKV infection has also been reported [33,53]. Thus, ZIKV infection, particularly early during pregnancy, can have devastating impacts on the infant, with the potential for life-long implications for both infants and their families.

In order to determine the impacts of ZIKV infection during pregnancy on early life, one longitudinal study tracked infants from birth to 18 months old [51]. They observed that at 12 months old, all infants diagnosed with CZS at birth demonstrated clear motor abnormalities and developmental delays, which persisted at follow-up six months later [51]. Similarly, Nielsen-Saines et al. evaluated neurodevelopment and neurosensory symptoms in a study of 216 infants born to mothers who had a rash and positive ZIKV RT-PCR in either serum or urine during pregnancy [52]. They found that while only eight infants were born with fetal microcephaly, 62 of 216 (28.7%) had abnormal neurodevelopmental assessments [52]. Furthermore, of 146 infants who were given Bayley Scales of Infant and Toddler Development, third edition assessments, 59 (40%) scored at least one standard deviation below average in at least one functional domain (language was the most affected functional domain) [52]. Perhaps the most striking aspect of these two studies was the observation that six infants (Vianna et al.) and two infants (Nielsen-Saines et al.) developed microcephaly after birth, despite being born without indications of CZS [51,52]. Together these studies strongly suggest that even infants born with no obvious brain abnormalities following ZIKV exposure during pregnancy are also at risk for neurodevelopmental delays or even post-natal CZS (Figure 1).

Several studies have also characterized ZIKV pathogenesis in pediatric cases of ZIKV infection. In one case report, a seven-year-old boy in Brazil presented with vomiting, headache, and tonic-clonic seizures, but no classic symptoms of ZIKV infection [55]. Upon admission to the ICU, he developed progressive weakness and areflexia, both of which resolved over several days, and which correlated with ZIKV RNA and infectious ZIKV particles in his CSF [55]. Similar CNS involvement was observed in a 15-year-old girl with ZIKV RNA in her CSF, who presented with left side paralysis and paresthesia (tingling sensation) seven days after developing left arm pain, headache, and conjunctival hyperemia (excess of blood in the blood vessels) [56]. More typical presentations were described in a prospective cohort study of 50 children aged 1–11 years and 90 adolescents aged 12–17 years [57]. Similar to what is typically described in adult ZIKV infection, maculopapular rash and fever were present in the majority of patients (94% and 74%, respectively), while 48% presented with arthralgia, 36% with conjunctivitis, and approximately 1 in 4 patients had gastrointestinal or respiratory involvement [57]. The authors noted that symptoms like arthralgia and myalgia were more common in adult cases than pediatric cases, and more common in 12–17-year-old patients than the 1–11-year-old patients, although the implications of this observation are unclear [57]. Nonetheless, these studies demonstrate that apart from rare cases with CNS involvement, ZIKV infection in children largely mirrors infection in adults.

## 4. Conclusions 

Following its initial isolation in 1947, ZIKV remained relatively unknown and innocuous until the Yap Island outbreak in 2007. Although no outbreaks of similar magnitude have been reported since the South and Central American outbreak, this and the outbreaks that preceded it have defined ZIKV as a pathogen with the clear capacity to cause wide-spread disease. This risk remains present as further small outbreaks continue to be reported, including in Singapore in 2016, India in 2018, and the recent retrospective identification of an outbreak in Cuba in 2017 [58,59,60]. Combined with the severe neurological symptoms associated with infection, ZIKV has clearly emerged as a major global health threat. In the context of pregnancy, this is especially true, as even children born with no obvious abnormalities may still be affected. While it cannot be definitively stated, it seems likely that these characteristics are newly acquired, as neither early case reports nor descriptions of the Yap Island outbreak indicated that ZIKV caused more than acute febrile illness. However, recent outbreaks have also occurred in the context of the introduction of ZIKV to novel host populations, which raises the possibility that host genetics, host nutritional habits, or previous immunity against other *flaviviruses*, among other factors, could contribute to increased epidemic capacity or pathogenesis. Nonetheless, the neurological symptoms that remain associated with ZIKV infection merit continued research efforts to understand risk factors associated with these symptoms and to inform vaccination or therapeutic approaches that are best-suited to prevent and treat severe ZIKV pathogenesis.
